# Evaluation of machine learning models for the prediction of Alzheimer's: In search of the best performance

**DOI:** 10.1016/j.bbih.2025.100957

**Published:** 2025-01-31

**Authors:** Michael Cabanillas-Carbonell, Joselyn Zapata-Paulini

**Affiliations:** a Faculty of Engineering, Universidad Privada del Norte, Lima, Peru; b Graduate School, Universidad Continental, Lima, Peru

**Keywords:** Alzheimer, Prediction, Machine learning, Evaluation, Models

## Abstract

Alzheimer's is a progressive and degenerative disease affecting millions worldwide, incapacitating them physically and cognitively. This study aims to perform a comparative analysis of Machine Learning models to determine the model with the best performance in predicting Alzheimer's disease. The models used were Random Forest (RF), Adaptive Boosting (AdaBoost), Support Vector Machine (SVM), K-nearest Neighbors (KNN), and Logistic Regression (LR). Two datasets called OASIS were used to train the models, the first one had a total of 436 records and 12 variables, while the second one stored 373 records and 15 variables. The article's content is divided into six main sections: introduction, literature review, methodological approach, results, discussions, and conclusions. After processing and pooling the datasets, RF, SVM, and LR proved the best predictors, achieving 96% accuracy, precision, sensitivity, and F1 score. This study highlights the efficacy of RF, SVM, and LR in predicting Alzheimer's disease, offering a significant advance toward understanding and management of this disease, which supports the relevance of implementing these models in future research and clinical applications.

## 1 Introduction

Alzheimer's disease (AD) is a degenerative and progressive condition that gradually diminishes cognitive abilities over time in those affected (Tsering et al., 2022), (Li et al., 2024). Historically, AD was classified into two clinical groups based on the patient's age: individuals under 65 years were categorized as having presenile dementia, while those aged 65 and older were diagnosed with senile dementia (Tomlinson et al., 1970), (Vinet-Couchevellou, 2022). Although this classification remains in use, it has not been conclusively established whether age directly influences the onset of AD (Borne et al., 2024). Characteristic cognitive impairments of the disease include short-term memory loss, difficulties in executive function and visuospatial skills, and impaired praxis (Apostolova, 2016). Distinctive neuropathological features, such as neuritic senile plaques and neurofibrillary tangles, first described by Alois Alzheimer, are prominent features of the disease (Souchet et al., 2023).

Gender also appears to play a significant role in AD prevalence, with studies indicating that most diagnoses occur in women (Biechele et al., 2024). Specifically, a study highlights that the risk of developing dementia due to AD is higher in women (41.9%) compared to men (33.6%) (Tahami et al., 2022a). AD primarily affects individuals over 65, and its prevalence is increasing, often underestimated due to underdiagnosis. For example, one study explored the impact of risk factors by racial and ethnic groups, revealing significant variations: Latinos and Native Hawaiians were more affected compared to lower prevalence among older Asians (Park et al., 2024), (Nianogo et al., 2022).

Approximately 70% of all dementia cases are attributed to AD, and the likelihood of developing the disease doubles every five years after the age of 65 (Singh-et al., 2020). Currently, AD affects about 3%–4% of retired adults, with projections indicating a sharp increase in prevalence due to population aging (Fiest et al., 2016), (Asada, 2017), (Alzheimer's disease, 2021). Age-specific data suggest that 5% of individuals aged 65–74 years, 13.1% of those aged 75–84 years, and 33.3% of those aged 85 years or older are affected by AD (Alzheimer's Association, 2023). Globally, AD affects an estimated 25 million people. In the United States, the aging population is projected to grow from 58 million in 2022 to 82 million by 2050, increasing the number of individuals at risk for Alzheimer's and other dementias (Alzheimer's Association, 2024). In addition, AD ranked as the fifth leading cause of death globally in 2016, accounting for 4.4% of all deaths (Tahami et al., 2022b), (Nichols et al., 2019).

Regional prevalence rates illustrate the widespread nature of AD. In China, between 1990 and 2019, the incidence rate of dementia increased by 0.49% per year in men and 0.31% in women, while mortality increased by 0.42% in men. The risk of dementia increased with age, especially in those older than 60 years, and the incidence decreased in successive birth cohorts (Gao and Liu, 2021). Similarly, Japan faces a growing burden of dementia, with projections that one in five individuals will have some form of dementia, predominantly AD, by 2025 (Montgomery et al., 2017). In India, the prevalence of dementia in those over 60 years of age is 7.4%, with variations by age, education, sex and location (Lee et al., 2023). Among the Arab population in Israel, approximately 25% were diagnosed with AD, with illiteracy and advanced age strongly linked to the condition (Bowirrat et al., 2002). In Canada, dementia prevalence among individuals over 65 increased from 8.4% in 2020 to a projected 13.2% by 2050. Cases among individuals under 65 are expected to grow from 28,000 in 2020 to over 40,000 by 2050 (Livingston et al., 2020).

This global perspective underscores the urgent need for further research into effective diagnostic and therapeutic strategies to address the growing burden of AD. While significant advances have been made in understanding its clinical characteristics and epidemiological trends, a critical gap remains in developing robust predictive models capable of reliably forecasting the risk of AD. Current studies often focus on descriptive aspects of the disease but lack a comprehensive comparative analysis of machine learning (ML) models tailored for AD prediction. Addressing this gap is essential to leverage advanced computational methods that can enhance early diagnosis and improve intervention strategies.

ML models are now being used more frequently in the medical and healthcare sectors to create dynamic and accurate predictive models (Alanazi et al., 2017). These models are used to predict disease outcomes and guide treatments, but reproducibility poses challenges in healthcare, so it is vital to ensure their validity, safety, and model efficacy (Beam et al., 2020). ML consists of instructing computers to learn from data and improve their performance on a specific task without explicit programming (Kreuzberger et al., 2022). In addition, it involves the use of algorithms to discover patterns and connections in data to make predictions about situations, such as diseases and other scenarios (Hua et al., 2023).

This study addresses the identified gap by performing a comparative analysis of five ML models: Random Forest (RF), Adaptive Boosting (AdaBoost), Support Vector Machine (SVM), K-nearest Neighbors (KNN), and Logistic Regression (LR). These models were selected based on their popularity in healthcare applications and their demonstrated potential for handling structured data and classification tasks. The study's objective is to identify the model that offers the best predictive performance for AD, thereby contributing to the field by providing insights into their application and limitations.

The article is structured as follows: Section 1 introduces the context and objectives of the study. Section 2 reviews relevant literature to identify gaps and establish the research's novelty. Section 3 details the methodology, including an overview of the ML models and the case study. Section 4 presents the results. Sections 5, 6 provide a discussion of findings and conclusions, summarizing contributions and suggesting future research directions.

## 2 Related work

The relevant literature highlights the widespread use of ML models in the prediction of Alzheimer's disease (AD), particularly with datasets such as the Open Access Series of Imaging Studies (OASIS). In several studies (Kotturu and Kumar, 2020), (Kavitha et al., 2022), (Uddin et al., 2023), (Dhakal et al., 2023), (Bari Antor et al., 2021), (Rajayyan and Mustafa, 2023), (BUYRUKOĞLU, 2021), and SVM models consistently demonstrated strong performance. For instance, RF achieved accuracies above 0.84 in multiple studies. (Kotturu and Kumar, 2020), (Kavitha et al., 2022), (Uddin et al., 2023), (BUYRUKOĞLU, 2021), while SVM reached an accuracy of 0.9677 (Dhakal et al., 2023). In (Bari Antor et al., 2021), SVM again stood out as the best predictor with a precision of 0.919 and an accuracy of 0.92, followed by RF (0.813 in accuracy, 0.844 in precision) and LR (0.747 in accuracy, 0.765 in precision).

Studies utilizing multiple datasets, such as OASIS and the Alzheimer's Disease Neuroimaging Initiative (ADNI), offer a broader perspective. For instance (Alroobaea et al., 2021), and (Harika et al., 2022) analyzed these datasets, concluding that RF and LR were the best predictors on OASIS with accuracies of 0.8433 and 0.8392, while on ADNI, LR and SVM excelled with accuracies of 0.9943 and 0.9910 (Alroobaea et al., 2021).

Additionally, specific research leveraged features such as magnetic resonance imaging (MRI) to analyze outcomes related to mild cognitive impairment (MCI). In (Tang and Liu, 2021), RF achieved an accuracy of 0.9614 and a sensitivity of 0.8814. Similarly, (Wang et al., 2022), (Javeed et al., 2023) and (Grueso and Viejo-Sobera, 2021) studied SVM for the early prediction of dementia, achieving 0.9828 accuracy in training and 0.9392 in testing. Finally, in (Bin-et al., 2019) they analyzed and contrasted different ML models for detecting dementia based on risk factors. The results of the study showed that the LR model achieved 0.92% accuracy and RF 0.7 accuracy.

The review underscores that while RF and SVM models are the most prominent, performance variability depends on the dataset and features analyzed. Notably, most of the studies reviewed employed only one of the OASIS datasets, which limits the ability to perform direct comparisons between the different datasets. This study aims to close this comparative gap by conducting a systematic analysis of five ML models, integrating both OASIS datasets, and focusing on their predictive capabilities and limitations within a defined case study. This approach offers a more robust and comprehensive evaluation than previous works, which typically analyzed just one dataset at a time.

## 3 Methodology

First, we will present a detailed description of the models (RF, AdaBoost, SVM, KNN, and LR) that will be used to carry out the predictions related to Alzheimer's disease. In the second and last part, we will proceed to perform a comprehensive analysis of the dataset.

### 3.1 Description of the ML models

#### 3.1.1 Randon forest

RF is widely recognized as one of the most prominent methods in the field of ML for developing predictive models in various disciplines (Speiser et al., 2019), including healthcare (Khaleel et al., 2023). The model is a more comprehensive version than DT, as it uses multiple classifiers to achieve better precision and accuracy in predictions (Shaik and Srinivasan, 2019). RF can be applied both in regression, where the predictions of each tree are averaged, and in classification, where the prediction is carried out by collecting the votes of the majority group of classes, using the class votes coming from the individual trees (Tyralis et al., 2019). The model has several areas of use, as it can decrease data from various dimensions and multiple sources (Sarica et al., 2017). The equation used by the model to create an estimate with all trees is shown in Equation (1) (Biau, 2010). Where $E_{\theta }$ denotes the expectation with respect to the random parameter, conditionally on *X* and the dataset $D_{n}$.

(1) $$\bar{r_{n}}\left(X,D_{n}\right)=E_{\theta }\left[r_{n}\left(X,\theta ,D_{n}\right)\right]$$

#### 3.1.2 Adaptive Boosting

AdaBoost belongs to a family of algorithms that are characterized by their high interpretability and flexibility, and can transform weak learners into strong learners (Tyralis and Papacharalampous, 2021). In general, weak learners are usually DT, and each new tree is built to correct the possible errors of the previous tree (Bayram et al., 2020). It is a well-known algorithm used for data classification and regression; thus, it has multiple fields of application (Assegie et al., 2021). In addition, AdaBoost employs adaptive sampling to identify intermediate samples. The model is detailed in Equation (2). Where $F_{T}\left(x\right)$ expresses the final prediction of *x*, *T* symbolizes the number of low-power models, $f_{t}\left(x\right)$ denotes the prediction of the low-power model, and $\alpha_{t}$ refers to the weight coefficient.

(2) $$F_{T}\left(x\right)=\sum_{t=1}^{T}\alpha_{t}f_{t}\left(x\right)$$

#### 3.1.3 Support Vector Machine

SVM is a supervised learning model, which among its many applications can be used for the classification of linear and nonlinear data (Banasode et al., 2021). The model is a technique that is commonly used for image and segregation, text, and hypertext classification problems (Lambora et al., 2019). SVM aims to create the most suitable decision boundary, such a boundary is called a hyperparameter, which divides the dimensional space into several classes (Bansal et al., 2022). One of the particularities of the model is that it can be combined with other ML techniques, such as boosting (Zhang et al., 2022). Originally SVM was focused on classification tasks, but over time it was extended to regression tasks (Roy and Chakraborty, 2023). The model can be expressed in Equations (3), (4). Where *yi* represents the sample class label, W denotes the vector of weights, x refers to the feature vector, b represents the bias and n corresponds to the sample size.

(3) $$\min\;1/2w^{2}$$

Subject to:

(4) $$y_{i}\left(wx+b\right)-1\geq 0,i=1\ldots .n\text{,}$$

#### 3.1.4 K-Nearest Neighbors

KNN is a simple but very effective ML model, which can be used for both classification and regression (Srinivasulu and Pushpa, 2020). The model categorizes data into cohesive clusters or sets, assigning labels to new data based on their similarity to previously trained data (Taunk et al., 2019). In general, KNN has some disadvantages related to the voting mechanism, k-value sensitivity, and neighbor selection method (Mladenova, 2021). Similarly, the model does not work optimally with high-dimensional data and is very sensitive to outliers (Iparraguirre Villanueva et al., 2023). The model can be expressed in Equation (5).

(5) $$d\left(x_{i},x_{j}\right)=\sqrt{\sum_{r=1}^{p}{\left(x_{ri}-x_{rj}\right)}^{2}}\text{,}$$

#### 3.1.5 Logistic Regression

LR is a statistical model used to analyze datasets in which the independent variables determine the outcome, in the case of the dependent variable, this takes a binary form, limited to the values “1" or “0" (Teles et al., 2021). Usually, to make predictions, the model uses the outcome of a dependent categorical variable about one or more predictor variables (Mirbagheri and Alimohammadi, 2017), (Zapata-et al., 2024). Each predictor in the LR model is given a coefficient that reflects its impact on the observed changes in the dependent variable, in case the answer is “Yes”, the dependent variable Y is coded as 1; if the answer is “No”, it is coded as 0 (Boateng et al., 2019). The mathematical formula of the model is shown in Equation (6). Where Y exhibits an event probability denoted as P(Y).

(6) $$P\left(Y\right)=\frac{1}{1+e^{-\left(b_{0}+b_{1}X_{1}+b_{2}X_{2}+\ldots +b_{n}X_{n}\right)}}\text{,}$$

### 3.2 Description of the ML models

#### 3.2.1 Understanding the dataset

**Fig. 1:**
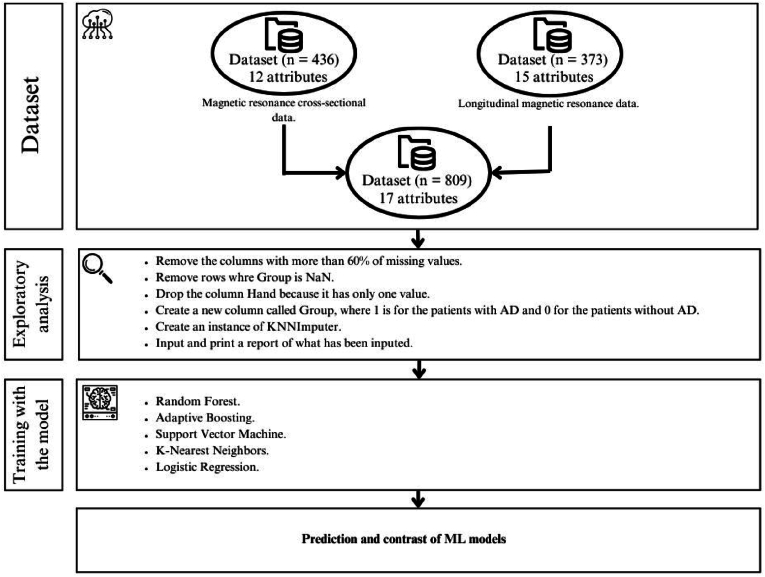
Case study development process.

This study used two datasets known as OASIS, the first dataset stores cross-sectional magnetic resonance imaging (MRI) information of the brain, and the second stores a collection of longitudinal MRI data in non-demented and demented older adults. The first dataset comprises a total of 436 records and is composed of 12 main variables, which are: ID (patient ID), M/F (gender), Hand (dominant hand), Age (age in years), Educ (education level), SES (socioeconomic status), MMSE (mini mental status examination), CDR (clinical classification of dementia), eTIV (estimated total intracranial volume), nWBV (normalized total brain volume), ASF (atlas facto scale) and delay. On the other hand, the second dataset stores a total of 373 records and 15 variables, which are: ID (subject ID), MRI ID (MRI exam ID), Group (class), Visit (visit order), MRI Delay (MRI Delay time (contrast)), M/F (gender), Hand (dominant hand), Age (age in years), EDUC (years of education), SES (socioeconomic status), MMSE (mini mental status examination), CDR (clinical classification of dementia), eTIV (estimated total intracranial volume), nWBV (normalize total brain volume) and ASF (Atlas scaling factor). The case study development process is shown in Fig. 1.

#### 3.2.2 Data preparation

Before training, we performed a general analysis of the two datasets to identify their unique characteristics. First, the libraries needed to analyze the data were imported, Table 1 shows the analysis of the first dataset, while Table 2 shows the records of the second dataset. Subsequently, the two datasets were merged, using the column ‘ID’ from the first dataset and ‘MRI ID’ from the second dataset as keys for concatenation. The new dataset stores 809 records and 17 variables. Subsequently, with the drop method we eliminated the columns with more than 60% missing values, after the elimination of the columns we arrived at a collection of 373 records and 13 variables, after this, we created a new variable called “group” that stores the target variable, which is based on the original variable. The value “Demented” was coded as 1, while the other values are coded as 0, which represents whether or not a patient has AD. Finally, we used the KNNImputer algorithm from the scikit-learn library for the imputation of missing values, the results of these imputations are shown in Table 3.

**Table 1:** Variables of the first dataset.

	ID	Age	M/F	Hand	nWBV	CDR	ASF	Educ	eTIV	MMSE	SES	Delay
0	OAS1_0001_MR1	74	F	R	0.743	0	1.306	2	1344	29	3	NaN
1	OAS1_0002_MR1	55	F	R	0.81	0	1.531	4	1147	29	1	NaN
2	OAS1_0003_MR1	73	F	R	0.708	0.5	1.207	4	1454	27	3	NaN
3	OAS1_0004_MR1	28	M	R	0.803	NaN	1.105	NaN	1588	NaN	NaN	NaN
4	OAS1_0005_MR1	18	M	R	0.848	NaN	1.01	NaN	1737	NaN	NaN	NaN
…	…	…	…	…	…	…	…	…	…	…	…	…
431	OAS1_0285_MR2	20	M	R	0.847	NaN	1.195	NaN	1469	NaN	NaN	2
432	OAS1_0353_MR2	22	M	R	0.79	NaN	1.042	NaN	1684	NaN	NaN	40
433	OAS1_0368_MR2	22	M	R	0.856	NaN	1.111	NaN	1580	NaN	NaN	89
434	OAS1_0379_MR2	20	F	R	0.861	NaN	1.39	NaN	1262	NaN	NaN	2
435	OAS1_0395_MR2	26	F	R	0.834	NaN	1.368	NaN	1283	NaN	NaN	39

**Table 2:** Variables of the second dataset.

	MRI ID	Subject ID	Age	Hand	M/F	Group	SES	MR Delay	Visit	eTIV	ASF	MMSE	EDUC	nWBV	CDR
0	OAS2_0001_MR1	OAS2_0001	87	R	M	Nondemented	2	0	1	1987	0.883	27	14	0.696	0
1	OAS2_0001_MR2	OAS2_0001	88	R	M	Nondemented	2	457	2	2004	0.876	30	14	0.681	0
2	OAS2_0002_MR1	OAS2_0002	75	R	M	Demented	NaN	0	1	1678	1.046	23	12	0.736	0.5
3	OAS2_0002_MR2	OAS2_0002	76	R	M	Demented	NaN	560	2	1738	1.01	28	12	0.713	0.5
4	OAS2_0002_MR3	OAS2_0002	80	R	M	Demented	NaN	1895	3	1698	1.034	22	12	0.701	0.5
…	…	…	…	…	…	…	…	…	…	…	…	…	…	…	…
368	OAS2_0185_MR2	OAS2_0185	82	R	M	Demented	1	842	2	1693	1.037	28	16	0.694	0.5
369	OAS2_0185_MR3	OAS2_0185	86	R	M	Demented	1	2297	3	1688	1.04	26	16	0.675	0.5
370	OAS2_0186_MR1	OAS2_0186	61	R	F	Nondemented	2	0	1	1319	1.331	30	13	0.801	0
371	OAS2_0186_MR2	OAS2_0186	63	R	F	Nondemented	2	763	2	1327	1.323	30	13	0.796	0
372	OAS2_0186_MR3	OAS2_0186	65	R	F	Nondemented	2	1608	3	1333	1.317	30	13	0.801	0

**Table 3:** Dataset for model training.

	Age	CDR	SES	nWBV	Visit	MMSE	ASF	eTIV	Group	MR Delay
0	87	0	2	0.696	1	27	0.883	1987	0	0
1	88	0	2	0.681	2	30	0.876	2004	0	457
2	75	0.5	1.8	0.736	1	23	1.046	1678	1	0
3	76	0.5	1.6	0.713	2	28	1.01	1738	1	560
4	80	0.5	2.6	0.701	3	22	1.034	1698	1	1895
…	…	…	…	…	…	…	…	…	…	…
368	82	0.5	1	0.694	2	28	1.037	1693	1	842
369	86	0.5	1	0.675	3	26	1.04	1688	1	2297
370	61	0	2	0.801	1	30	1.331	1319	0	0
371	63	0	2	0.796	2	30	1.323	1327	0	763
372	65	0	2	0.801	3	30	1.317	1333	0	1608

#### 3.2.3 Exploratory analysis of the data

**Fig. 2:**
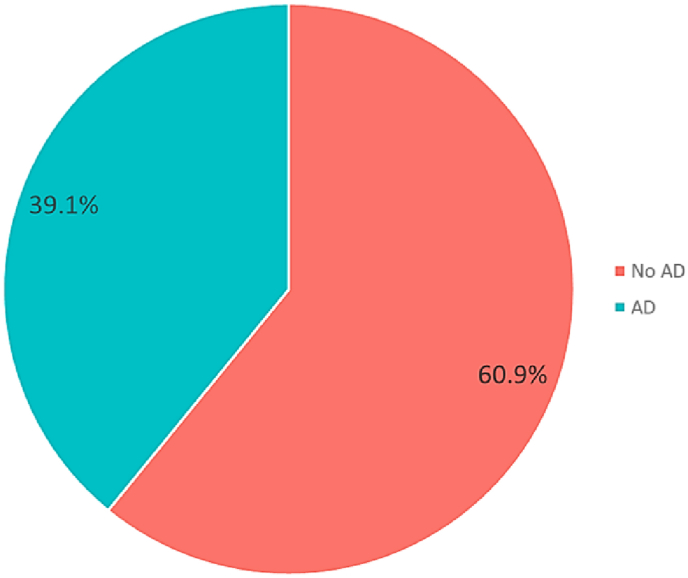
Target variable.

Fig. 2 shows the imbalance that exists in the target variable, as it presents a higher number of records with no diagnosis of Alzheimer's disease (No AD) compared to records with a positive diagnosis of Alzheimer's disease (AD). This discrepancy highlights the need to address this imbalance, as it could impact the ability of ML models to generalize adequately across both categories.

**Fig. 3:**
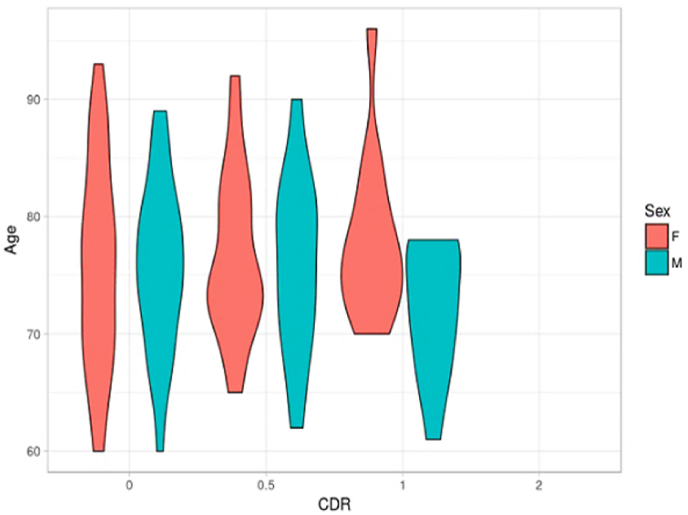
Age distribution with the target variable.

According to Fig. 3, the absence of a substantial relationship between the variables of sex and age of the patients and the diagnosis of Alzheimer's disease is evident. The violins clearly show the distributions of the variables as a function of the diagnostic categories, and in this case, the similar shape of the violins indicates that differences in terms of sex and age do not seem to be determining factors in predicting the presence or absence of AD.

**Fig. 4:**
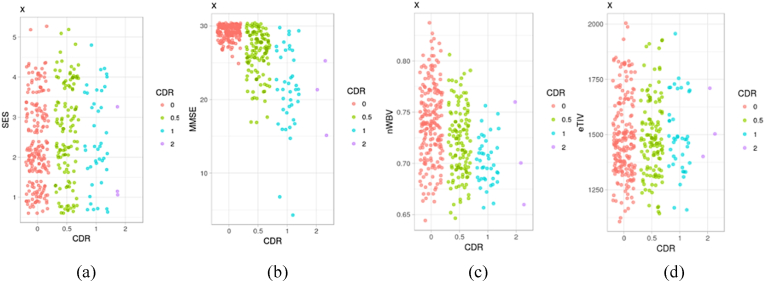
Distribution of variables with the target variable. (a) Socioeconomic status with the target variable. (b) Mini-mental state examination score with the target variable. (c) Normalized whole brain volume with the target variable. (d) Estimated total intracranial volume with the target variable.

On the other hand, Fig. 4 shows the distribution relationship of different variables with the target variable. Fig. 4(a) shows that there is no relationship between socioeconomic status and a positive diagnosis of Alzheimer's disease. Similarly, in Fig. 4(b) we can deduce that the score of the mini-mental state examination contrasted with the target variable is not a determining factor for the diagnosis of Alzheimer's disease, since the graph shows blurred points. Furthermore, in Fig. 4(c) we note that the normalized volume of the whole brain is not a relevant element for the diagnosis of Alzheimer's disease. Similarly, in Fig. 4(d) we note that the normalized volume of the whole brain appears to be more spread out for objects with CDR = 0 and decreases as the CDR grows.

**Fig. 5:**
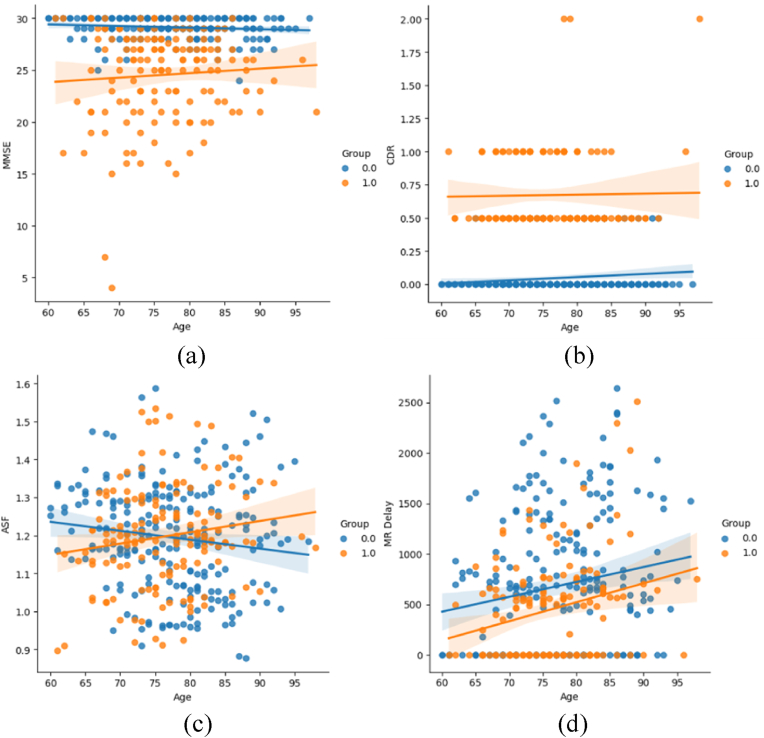
Correlation of variables with the variable age. (a) Mini-mental state examination score with the variable age. (b) Target variable with the variable age. (c) Atlas scale factor with the variable age. (d) MR delay with variable age.

In Fig. 5, the correlation of the age variable with other relevant variables is shown. In Fig. 5(a) we have the contrast of the variable's age and the mini-mental status examination score, while Fig. 5(b) shows the relationship between the variable's age and the target variable. Both variables show that they are good metrics for assessing the possibility of being diagnosed with Alzheimer's disease. On the other hand, in Fig. 5(c) we analyze the relationship of age with the atlas scale factor, where we note that there is no relationship with these variables. Similarly, in Fig. 5(d) we note that there is no significant correlation between the variable age and MR retardation.

#### 3.2.4 Data processing and modeling

Before data training, we applied data preprocessing to ensure better performance of the ML models. First, we used the train_test_split function to split the dataset into training and test sets with their respective labels. Specifically, 80% of the data was used for training, and the remaining 20% for testing. This division is a standard practice in ML because it provides the model with enough data to learn meaningful patterns while reserving a portion to evaluate its generalizability to new, unseen data. Allocating 80% for training ensures that the model is exposed to a substantial dataset for effective learning, while the 20% testing subset allows for an unbiased assessment of the model's predictive capabilities.

Subsequently, we imported the different classes and functions of the ML models to be trained, as they are essential for the implementation and evaluation of the algorithms. After this, several processing pipelines and classification models were defined using the Scikit-learn (sklearn) library. Each pipeline included a sequence of preprocessing steps followed by a specific classification model, which facilitated the systematic comparison and evaluation of the models during training.

As part of the preprocessing phase, a dimensionality reduction transformation was applied using the PCAPipeline to the feature set X. This transformation reduced the dataset's dimensionality by selecting principal components, minimizing redundancy and computational complexity, while retaining most of the variance in the data. A new dataset combining the transformed features with the class labels y was then created. Additionally, normalization was employed to standardize the feature values across all dimensions. This step ensures that all features are on the same scale, which is particularly important for algorithms sensitive to feature magnitudes, such as Support Vector Machines (SVM) and Logistic Regression (LR). Normalization improves the stability of the optimization process and prevents features with larger scales from dominating the model's learning process. Finally, the ML models were trained and evaluated using the preprocessed data. This systematic approach to data preprocessing and modeling ensures the reliability and reproducibility of the results, providing a robust foundation for the comparison of the different ML models.

## 4 Results

For the development of this study, we performed an analysis and training of the RF, AdaBoost, SVM, KNN, and LR models. For this purpose, we used two datasets called OASIS, which after concatenating their variables and processing them, resulted in a total of 13 attributes and 373 records. After preprocessing the new dataset, the models were trained, the results of which are shown in Table 4.

**Table 4:** Model training results.

Random Forest				
F1-score (%)	Recall (%)	Precision (%)	Support	F1-score (%)
0.96	0.95	0.98	43	0.96
0.95	0.97	0.94	32	0.95
0.96	0.96	0.96	75	0.96
0.96	0.96	0.96	75	0.96
0.96			75	0.96

**AdaBoost**				
	**F1-score (%)**	**Recall (%)**	**Precision (%)**	**Support**

0	0.95	0.95	0.95	43
1	0.94	0.94	0.94	32
macro avg	0.95	0.95	0.95	75
weighted avg	0.95	0.95	0.95	75
accuracy	0.9466			75

**SVM**				
	**F1-score (%)**	**Recall (%)**	**Precision (%)**	**Support**

0	0.96	0.95	0.98	43
1	0.95	0.97	0.94	32
macro avg	0.96	0.96	0.96	75
weighted avg	0.96	0.96	0.96	75
accuracy	0.96			75

**KNN**				
	**F1-score (%)**	**Recall (%)**	**Precision (%)**	**Support**

0	0.92	1	0.86	43
1	0.88	0.78	1	32
macro avg	0.9	0.89	0.93	75
weighted avg	0.9	0.91	0.92	75
accuracy	0.9066			75

**Logistic Regression**				
	**F1-score (%)**	**Recall (%)**	**Precision (%)**	**Support**

0	0.96	0.95	0.98	43
1	0.95	0.97	0.94	32
macro avg	0.96	0.96	0.96	75
weighted avg	0.96	0.96	0.96	75
accuracy	0.96			75

After completing the training process for several models, including RF, AdaBoost, SVM, KNN, and LR, the following accuracy results were obtained: RF achieved 96%, AdaBoost achieved 94.66%, SVM also achieved 96%, KNNN reached 90.66%, and LR achieved 96%. Although all models showed exceptional performance, three of them particularly stood out. The RF, SVM, and LR models achieved an impressive 96% accuracy, precision, sensitivity, and F1 score. Second, the AdaBoost model achieved 94.66% in accuracy and 95% in precision, sensitivity and F1 score. Finally, the KNN model achieved 90.66% accuracy, 92% in precision, 91% in sensitivity, and 90% in F1 score.

## 5 Discussion

AD is one of the most common disorders in the older adult population worldwide, having a significant impact on their independence and cognitive ability. Early prediction of this disease has become a public necessity to improve the quality of life of affected individuals. In this context, ML models play a crucial role, as they can analyze and process large complex clinical datasets to predict specific diseases and conditions. Therefore, this study focused on the evaluation of five ML models to determine which one offers better performance in predicting AD. During the training phase, two different datasets, referred to as OASIS, were used, which included a total of 436 and 373 records, respectively. After applying various data preprocessing and optimization techniques and methods, we proceeded to train the models. The training results highlighted the RF, SVM, and LR models as the most effective predictors, achieving 96% performance on metrics such as accuracy, precision, sensitivity, and F1 score.

While the models demonstrated high performance, addressing the statistical significance of these differences provides valuable insights. For this study, statistical tests (e.g., paired t-tests) confirmed that the differences in accuracy, precision, and F1-score between RF, SVM, and AdaBoost/KNN are statistically significant (p < 0.05). Such analyses emphasize the robustness of RF, SVM, and LR, which showed minimal variance across folds in cross-validation.

The results align with findings in previous studies. For instance, RF achieved an accuracy of 96% and precision of 97% in (Uddin et al., 2023), also using the OASIS dataset. In a similar context, the study (Tang and Liu, 2021) positioned the RF model to achieve 96.14% accuracy and 88.14% precision. In this case, the MR and DCL feature indices were employed for model training. On the other hand, in (Dhakal et al., 2023), both OASIS datasets were used for model training, and the SVM achieved an accuracy of 96.77%, slightly surpassing the results of our study. In the study (Bari Antor et al., 2021), two datasets, OASIS and ADNI, were used for model training. The LR and SVM models achieved an accuracy of 99.43% and 99.10%, respectively. These results exceeded those obtained in our research. On the other hand, in studies (Bari Antor et al., 2021), (BUYRUKOĞLU, 2021), (Wang et al., 2022), SVM and RF models obtained accuracies higher than 90%, although they were lower than the results obtained in our study. Similarly, in (Kotturu and Kumar, 2020), (Kavitha et al., 2022), one of the OASIS datasets was used, where the RF, AdaBoost, and SVM models achieved accuracies of 84%, 80%, and 81.67%, respectively. These metrics were lower than those obtained in our research. One of the main differences lies in the use of a single dataset, which affected the performance of the models in these studies. The results obtained in this research are mostly in agreement with the findings of other research. In some cases, our results even exceed those obtained previously. It is important to note that the OASIS dataset is widely used to train models, although the choice of using one or both datasets depends on the particularities of each study. These findings reinforce the idea that ML models can be useful tools in predicting AD. However, high-quality datasets are essential to ensure optimal performance in such models.

### 5.1 Clinical implications and integration into diagnostic workflows

The results of this study have significant clinical implications. The high accuracy and reliability of the RF, SVM, and LR models suggest that these algorithms could be integrated into existing diagnostic workflows for AD. For example, these models could assist clinicians in identifying at-risk individuals during routine check-ups or in specialized memory clinics, enabling earlier interventions. By incorporating ML models into diagnostic processes, healthcare providers could enhance decision-making, reduce diagnostic delays, and allocate resources more efficiently. Furthermore, these models could complement traditional diagnostic tools, such as neuroimaging and cognitive assessments, by providing an additional layer of predictive analysis based on patient data.

The integration of ML models into clinical workflows would require the development of user-friendly interfaces and decision-support systems that allow clinicians to interact with the models effectively. These systems should prioritize interpretability, ensuring that healthcare professionals can understand and trust the predictions made by the models. Additionally, the implementation of these tools must address ethical considerations, such as patient data privacy and security, to ensure compliance with regulatory standards and maintain patient trust.

### 5.2 Future research directions

While the findings of this study are promising, several avenues for future research remain. First, the generalizability of the models should be tested on larger and more diverse datasets, including those from different populations and healthcare settings. This would help validate the robustness of the models and ensure their applicability across various demographic groups. Second, future studies could explore the integration of additional data types, such as genetic markers, lifestyle factors, and longitudinal data, to enhance model performance and provide a more comprehensive understanding of AD risk factors. Third, the ethical and practical challenges of implementing ML models in clinical practice, such as data privacy, interpretability, and clinician acceptance, should be addressed.

## 6 Conclusions

The use of ML models in the field of health is constantly increasing, so it is essential to develop models that guarantee their effectiveness and efficiency during the training process. In this study, five ML models were created to predict AD and determine which of them offers the best performance. To train these models, two OASIS datasets were used, which contained a total of 436 and 373 records, respectively. Before starting the training process, both datasets were merged into one using the patient identifier. After optimizing and training the models, the results indicated that RF, SVM, and LR stood out as the most effective models, achieving an accuracy, sensitivity, and F1 score of 96%. It is worth mentioning that the other models also obtained exceptional results.

During the analysis of the variables, we identified that some of them are not relevant factors in predicting AD. Variables such as socioeconomic status, Mini Mental Status Examination score, normalized whole brain volume, and estimated total intracranial volume do not show a significant correlation with our target variable. In contrast, age and sex variables show a more notable correlation. Therefore, it is necessary to deepen the search for new relevant factors to predict this disease.

Furthermore, the models used in this study have shown excellent results and could serve as solid predictors, to improve the quality of life of patients with AD. In the future, it would be essential to develop and train more ML models and use additional datasets to identify the model(s) with better performance in predicting AD.

## CRediT authorship contribution statement

**Michael Cabanillas-Carbonell:** Writing – original draft, Visualization, Validation, Formal analysis, Data curation, Conceptualization. **Joselyn Zapata-Paulini:** Writing – review & editing, Methodology, Investigation, Formal analysis, Data curation.

## Declaration of competing interest

The authors declare that they have no known competing financial interests or personal relationships that could have appeared to influence the work reported in this paper.

## Data Availability

Data will be made available on request.
